# Continuous Flow Separation of Red Blood Cells and Platelets in a Y-Microfluidic Channel Device with Saw-Tooth Profile Electrodes via Low Voltage Dielectrophoresis

**DOI:** 10.3390/cimb45040200

**Published:** 2023-04-04

**Authors:** Rodward L. Hewlin, Maegan Edwards

**Affiliations:** 1 Center for Biomedical Engineering and Science (CBES), Department of Engineering Technology and Construction Management (ETCM), University of North Carolina at Charlotte, Charlotte, NC 28223, USA; 2 Applied Energy and Electromechanical Systems (AEES), Department of Engineering Technology and Construction Management (ETCM), University of North Carolina at Charlotte, Charlotte, NC 28223, USA

**Keywords:** cells, computational, dielectrophoresis, hydrodynamic focusing, microfluidics, multiphysics, platelets, particle separation, particle trajectory, red blood cells

## Abstract

Cell counting and sorting is a vital step in the purification process within the area of biomedical research. It has been widely reported and accepted that the use of hydrodynamic focusing in conjunction with the application of a dielectrophoretic (*DEP*) force allows efficient separation of biological entities such as platelets from red blood cell (*RBC*) samples due to their size difference. This paper presents computational results of a multiphysics simulation modelling study on evaluating continuous separation of RBCs and platelets in a microfluidic device design with saw-tooth profile electrodes via DEP. The theoretical cell particle trajectory, particle cell counting, and particle separation distance study results reported in this work were predicted using COMSOL v6.0 Multiphysics simulation software. To validate the numerical model used in this work for the reported device design, we first developed a simple y-channel microfluidic device with square *“in fluid”* electrodes similar to the design reported previously in other works. We then compared the obtained simulation results for the simple y-channel device with the square in fluid electrodes to the reported experimental work done on this simple design which resulted in 98% agreement. The design reported in this work is an improvement over existing designs in that it can perform rapid separation of RBCs (*estimated 99% purification*) and platelets in a total time of 6–7 s at a minimum voltage setting of 1 V and at a minimum frequency of 1 Hz. The threshold for efficient separation of cells ends at 1000 kHz for a 1 V setting. The saw-tooth electrode profile appears to be an improvement over existing designs in that the sharp corners reduced the required horizontal distance needed for separation to occur and contributed to a non-uniform DEP electric field. The results of this simulation study further suggest that this DEP separation technique may potentially be applied to improve the efficiency of separation processes of biological sample scenarios and simultaneously increase the accuracy of diagnostic processes via cell counting and sorting.

## 1. Introduction

Platelet separation and purification are required in a variety of medical applications ranging from the detection and treatment of hemorrhagic and thrombotic diseases to blood transfusions [1,2]. Low platelet concentration can cause hemorrhaging, whereas high concentration can lead to thrombosis and related complications such as infarction, embolism, or stroke. As a result, it is vital to monitor platelet concentration to diagnose such unconventionalities early for appropriate diagnosis and treatment. To date, there remains a large demand for platelet sample preparation in applications such as blood transfusion or medical research. In an attempt to satisfy this need, there have been several techniques that have been investigated for such separation applications. One of the most common separation methods for biological samples is centrifugation [3,4]. However, the main drawback of centrifugation is the need for professional equipment, robust laboratory facilities, and operators, and the process is also expensive, labor intensive, and non-suitable for point-of-care testing [5,6,7].

Lab-on-a-chip (*LOC*) devices have been used in a variety of research applications for cellular manipulation and sorting involving cancer diagnosis [5,6], pathogen detection, and rapid genomic testing [7,8]. Immunolabeling, magnetic bead separation, and laminar flow-based separation are some of the most common techniques utilized in LOC applications. In addition to current methods for cellular manipulation at the micro-scale, methods based on magnetic bead targeting have been investigated at the macroscale level in computational and experimental work for potential medical drug targeting [9,10]. On the micro-scale level, separation methods based on magnetic beads require labeling, as well as multiple and prolonged incubation and wash cycles. Using functionalized magnetic beads to separate target molecules and cells could potentially overcome these challenges using magnetic fields as opposed to electric fields [11,12]. The drawback of this technique is the lengthy incubation times and wash cycles, as well as the difficulty of removing the label post priori [13].

Dielectrophoresis (*DEP*) has been widely reported as an exceptional technique for cell discrimination and isolation for biological sample processing, sorting of biological cells [14,15], droplets [16,17], and particles [17,18,19,20]. In a recent work done by Yamashita et al., this group established a method of high purification of platelets using DEP to eliminate blood cells from platelet concentrates [21]. Zhao et al. demonstrated the isolation of CTCs from blood cells by the combination of DEP and magnetophoresis in a microfluidic chip [22]. Li et al. [23] utilized DEP to separate live and heat-treated Listeria innocua cells. Gascoyne et al. [24] applied DEP to isolate malaria-infected cells from blood. Moon et al. [25] successfully separated human breast cancer cells (*MCF-7*) from a spiked blood cell sample by combining multi-orifice flow fractionation (*MOFF*) and DEP. Song et al. [26] utilized a continuous-flow microfluidic device based on the accumulation of multiple DEP forces to sort stem cells and their differentiation progeny at different flow rates. Wang et al. proposed a novel microfluidic chip for the continuous separation of microalgae cells based on AC DEP [27]. Vahey et al. demonstrated the separation of polystyrene beads based upon surface conductance as well as sorting non-viable from viable cells of the budding yeast Saccharomyces cerevisiae through DEP [28]. Cao et al. [29] demonstrated highly effective enrichment of proteins by using nanoscale insulator-based DEP (*iDEP*) integrated with Ag/SiO_2_ Nanorod Arrays. Kung et al. [30] utilized a tunnel DEP (*TDEP*) mechanism for continuously tunable, sheathless, 3D, and single-stream microparticle and cell focusing in high-speed flows.

One of the main disadvantages reported is in order to achieve a high separation resolution and enough throughput, high voltages are typically necessary to induce a strong DEP effect, which may induce Joule heating effects in the microchannel and limit their application for temperature-sensitive biological cells [31]. Similarly, the particle force produced must be higher that the hydrodynamic force and flow effects [32,33,34,35,36,37]. This paper presents computational results of a multiphysics simulation modelling study on evaluating continuous separation of RBCs and platelets in a microfluidic device with saw-tooth electrodes via DEP. The theoretical cell trajectory results reported in this work were predicted using COMSOL v6.0 Multiphysics software. The main contributions of this work are as follows.

1. The design reported in this work is an improvement over existing designs in that it can perform rapid separation of red blood cells (*estimated 99% purification*) of platelets in less than a total time of 6–7 s at a voltage setting of 1 V and at a minimum frequency of 1 Hz.
2. The presentation of an operating parameter optimization study on driving parameters such as frequency and voltage settings.
3. The results of the simulation study suggest that the saw-tooth electrode design appears to be an improvement over existing designs in that the sharp corners reduced the required horizontal distance needed for separation to occur and contributed to a non-uniform DEP electric field.
4. The results of this simulation study suggest that this DEP separation technique may potentially be applied to improve the efficiency of separation processes of biological sample scenarios and simultaneously increase the accuracy of diagnostic processes via cell counting and sorting.

The next section presents the methodology of this work.

## 2. Methodology

This section of the paper describes the fluid flow, electric field distribution, and DEP force modelling methodology for the multiphysics simulations. All multiphysics modelling was performed in COMSOL v6.0 Multiphysics software. The next section discusses the laminar microfluid flow modelling.

### 2.1. Laminar Microfluid Flow Modelling

In a microfluidic system such as the one proposed in this work, laminar fluid flow corresponds to the flow with a small Reynolds number (*Re*) where (*Re <* 1). The Reynolds number is a dimensionless number that compares fluid flow inertia to viscous terms. The viscous term of the Reynolds Number dominates in the case of the proposed work, which indicates that the inertia forces can be ignored. The physical formulation of the Navier–Stokes, energy, strain, and viscous stress tensor equations for single phase flow in the formulation that COMSOL uses are listed below [38]:

**Conservation of Mass and Continuity**:

(1) ρ∇⋅u=0

**Conservation of Momentum:**

(2) $$\rho \frac{\partial u}{\partial t}+\rho \left(u\cdot \nabla \right)u=\nabla \cdot \left[-PI+\tau \right]+F$$

**Conservation of Energy Equation:**

(3) $$\rho C_{P}\left(\frac{\partial T}{\partial t}+\left(u\cdot \nabla \right)T\right)=-\left(\nabla \cdot q\right)+\tau :S-{\left.\frac{T}{\rho }\frac{\partial \rho }{\partial T}\right|}_{P}\left(\frac{\partial P}{\partial t}+\left(u\cdot \nabla \right)P\right)+Q$$

**Strain Tensor:**

(4) $$S=\frac{1}{2}\left(\nabla u+{\left(\nabla u\right)}^{T}\right)$$

**Viscous Stress Tensor:**

(5) $$\tau =2\mu S-\frac{2}{3}\mu \left(\nabla \cdot u\right)I$$

where *ρ* is the density, *I* is the unit matrix, *u* is the velocity vector, *P* is the pressure, *τ* is the viscous stress tensor, *C_p_* is the specific heat capacity at constant pressure, *T* is the absolute temperature, *q* is the heat flux, *Q* is the heat source, *S* is the strain tensor, is, *µ* is the dynamic viscosity of the fluid, *T* is the temperature, and *F* is the body force.

For fluid flow modelling in both the simple square electrode design and the proposed design, the corresponding boundary conditions are as follows: (1) The microchannel walls are modelled with the no-slip boundary condition; (2) the inlet fluid velocity boundary condition for the sheath (*buffer*) flow is 134 μm/s and the velocity boundary condition for RBCs and platelets is 853 μm/s; and (3) the fluid at the outlet is modelled as a pressure outlet condition where the pressure is set to be atmospheric pressure, that is, *P* = 0 (*gauge*). Wall shear stress is evaluated using the expression:

(6) $$\tau_{w}=\mu \frac{\partial u}{\partial y}$$

### 2.2. Mesh Independence Evaluation Methodology

**Figure 1:**
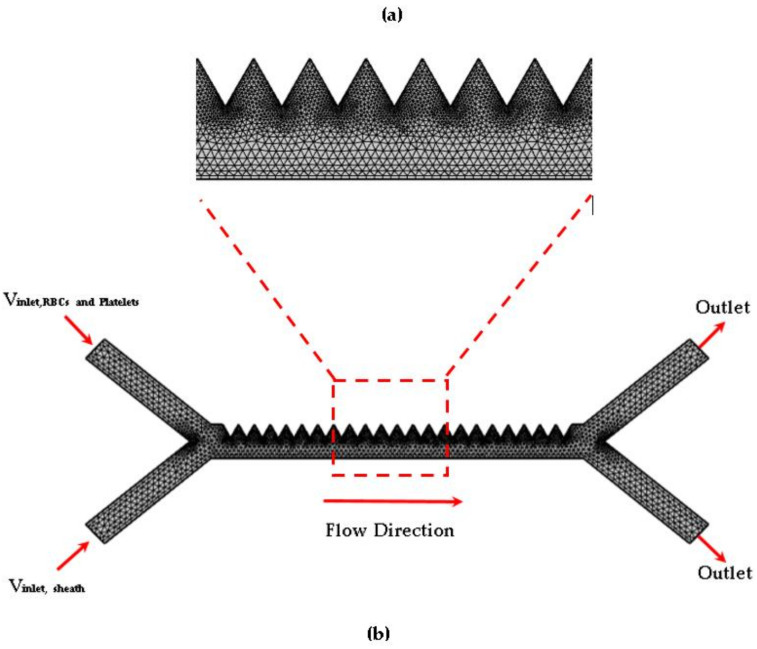
Selected computational mesh for the proposed design analysis: (**a**) zoomed in view and (**b**) full view.

To achieve mesh independence, a mesh independence study was conducted on both the simple square electrode and the proposed design to obtain the optimum mesh element size for running the simulations where the results would no longer be dependent on the mesh size. In this work, four types of mesh types were analyzed: Coarse, Fine, Extra Fine, and Finest. A velocity profile extraction was used to verify the mesh size independence and is discussed in the Section 3 (Section 3.2.1) of this paper. Figure 1 below shows a snapshot of the selected mesh with the prescribed boundary conditions. The next section describes the electric field modelling methodology.

### 2.3. Electric Field Modelling Methodology

For electric field simulations of both the simple square electrode design and proposed design, the electric fields were modeled under steady-state conditions. The electric field distribution within the micro-channel is described and modelled by the Laplace equation:

(7) $$\nabla \cdot J=Q_{i}$$

(8) $$J=\sigma E+J_{e}$$

(9) E=−∇V

where *J* is the current density, *E* is the electric field, *J_e_* is the external current density, *Q* is the power dissipation, and σ is the electrical conductivity. The next section presents the DEP modelling methodology.

### 2.4. Dielectrophoresis Theory

**Figure 2:**
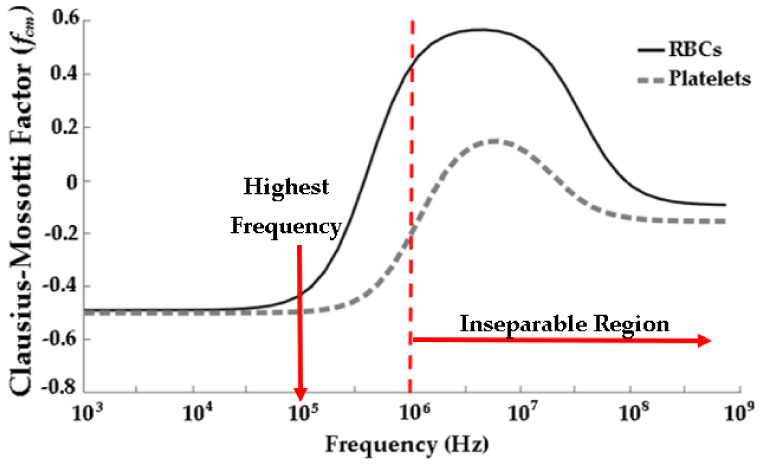
Plot of the real part of the Clausius–Mossotti factor for RBCs and platelets in a medium with a conductivity of 55 mS/m, using a single-shell model with the parameters found in the literature [41].

The DEP force is generated due to an induced non-uniform electric field subjected to particles within a micro-channel device purposed for DEP separation. The particles are polarized to generate corresponding induced charges, which in turn generates dipole moments [39]. The ends of the positive and negative charges of the particles are uneven due to the force which influences particle motion. In this work, the mechanism of particle separation was analyzed by using negative dielectrophoretic forces of RBCs and platelets. DEP is the movement of particles in a non-uniform electric field due to the interaction of the particle’s induced dipoles with the spatial gradient of the electric field norm. The DEP exerted on a spherical particle is generally expressed as [40]:

(10) FDEP=2πεmr3RefCM∇E2

where

(11) $$f_{CM}=\left(\frac{\varepsilon_{p}^{*}-\varepsilon_{m}^{*}}{\varepsilon_{p}^{*}+2\varepsilon_{m}^{*}}\right)$$

and

(12) $$\varepsilon^{*}=\varepsilon -j\frac{\sigma }{\varpi }$$

where *ε*_0_ and *ε_m_* are the vacuum permittivity and the permittivity of the suspending medium, and ε_m_* and ε_p_* are the complex permittivity of the suspending medium and the particle, respectively. *E* represents the root–mean–squared electric field strength, and Re(*f_CM_*) represents the real part of the Clausius–Mossotti *(CM)* factor. ω is the frequency of the applied electric field. ε and σ are the permittivity and conductivity of the material. The real part of the *CM* factor “Re(*f_CM_*)” varies between [−0.5, 1]. Figure 2 shows the real part of the Clausius–Mossotti factor for RBCs and platelets plotted against frequency.

**Figure 3:**
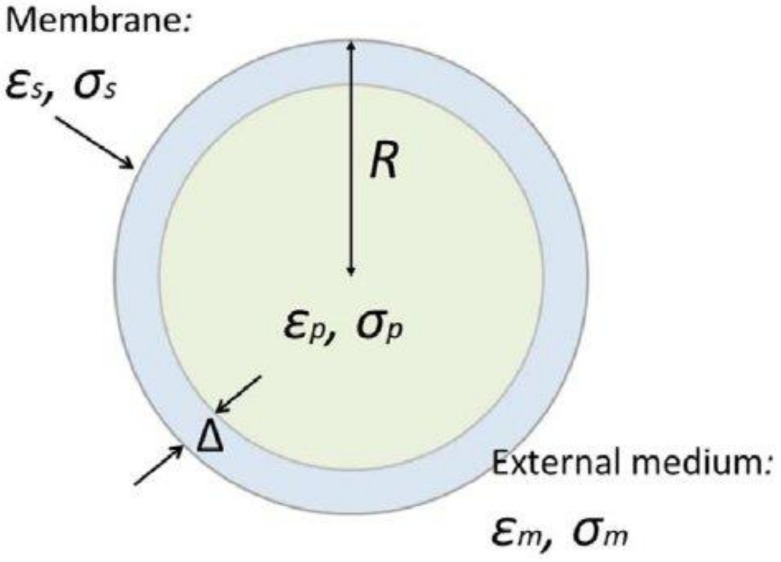
The single-shell model. A spherical particle representing a cell with radius *R*, permittivity ε_p_ and conductivity σ_p_, which is covered by a uniform layer of thickness Δ << *R*, permittivity ε_s_, and conductivity σ_s_. Obtained from the work of Chau et al. [42].

An RBC cell consists of cytoplasm surrounded by a membrane. RBCs and platelets can be considered a single-shell model rather than homogeneous spheres, as shown in the schematic provided in Figure 3 below.

In this work, we consider the simple single spherical model, whose effective dielectric constant is ε_eq_* [43]:

(13) $$\varepsilon_{eq}^{*}=\varepsilon_{s}^{*}\frac{{\left(\frac{r_{0}}{r_{i}}\right)}^{3}+\frac{2\left(\varepsilon_{p}^{*}-\varepsilon_{s}^{*}\right)}{\varepsilon_{p}^{*}+2\varepsilon_{s}^{*}}}{{\left(\frac{r_{0}}{r_{i}}\right)}^{3}-\frac{2\left(\varepsilon_{p}^{*}-\varepsilon_{s}^{*}\right)}{\varepsilon_{p}^{*}+2\varepsilon_{s}^{*}}}$$

where *r_0_* and *r_i_* are the outer and inner radii of the shell, respectively, ε_p_* is the complex permittivity of the particles, and ε_s_* is the complex permittivity of the outer shell. When calculating the DEP force, the complex permittivity *ε_p_* of the particle is replaced by the equivalent complex permittivity ε_eq_* of the uniform particle composed of the shell and the interior of the particle. The material and dielectric properties used in this work are listed in Table 1.

### 2.5. Cell Trajectory Modelling

The modeling of the particle flow is laminar, and the fluid is mainly affected by the hydrodynamic force. In this case, the inertial force is negligible. In the normal temperature and pressure environment, the force caused by Brownian motion is small and has very little effect on particle targeting and motion. In addition, the particle density is similar to the fluid density, and the particles will stay in the channel for a short period of time. The sedimentation of the particles in the vertical direction can be ignored. Therefore, the DEP force and fluid viscous force will be mainly considered when investigating the force movement of particles in this system. The trajectory of the particles is described as:

(14) $$m\overset{\cdot \cdot }{x}(t)+K_{f}\overset{\cdot }{x}(t)=F_{DEP}(x,E,t)$$

(15) $$K_{f}\overset{\cdot }{x}(t)=6\pi r\mu v$$

(16) $$\overset{}{x}(t)={\left[x,y\right]}^{T}$$

where *m* represents the mass of the particle and *x*(*t*) is the position of the particle in the vertical coordinate system. *K_f_ ẋ*(*t*) is the linear viscous resistance and ν is the kinematic viscosity. The next section presents the device geometric modelling.

### 2.6. Device Geometric Modelling

**Figure 4:**
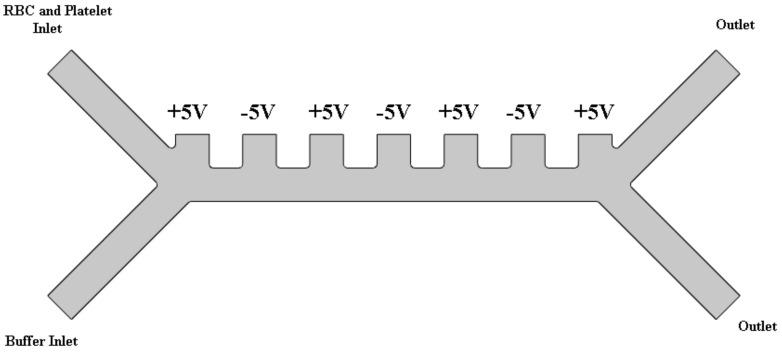
Simple square in fluid electrode microfluidic design.

There are two device geometries modelled and evaluated in this work. The first is the simple square in fluid electrode design. The simple square in fluid electrode design evaluated in this work is based on a lab-on-a-chip device described in detail in the work of Piacentini et al. [44] and Tornay et al. [45]. This model consists of two inlets, two outlets, and a separation region in which a non-uniform electric field created by an arrangement of electrodes of alternating polarity alter the particle trajectories. The channel dimensions are 40 µm in height and 625 µm in length. The electrode dimensions are 45 µm × 45 µm with a 45 µm spacing. Figure 4 shows the schematic of the modeled geometry. As shown in Figure 4, the inlet velocity for the lower inlet for buffer is significantly higher (853 μm/s) than the upper inlet (154 μm/s) in order to focus all the injected particles toward the upper outlet.

**Figure 5:**
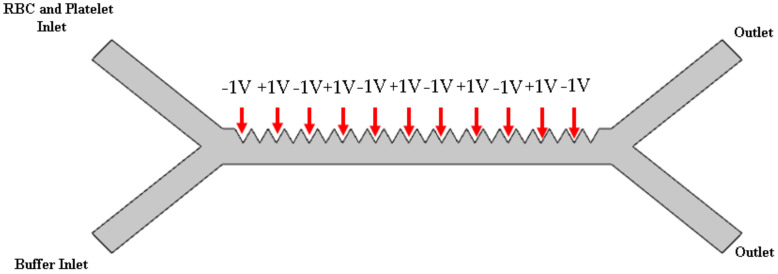
Proposed microfluidic design with saw-tooth electrode profile.

A schematic of the proposed microfluidic design is shown in Figure 5. The inlets have the same boundary conditions as the simple square in fluid electrode design. The channel dimensions for the proposed design are 50 µm in height and 625 µm in length. The electrode dimensions are 20 µm base × 20 µm height with a 20 µm non-electrode spacing. The red arrows in Figure 5 show where the voltages are applied.

The next section discusses the conclusions of this work.

## 3. Results

### 3.1. Model Validation Results Via Comparison to Previous Works

This section of the paper presents the results of the numerical simulations of the proposed design. In this design analysis, we first begin with presentation of the validation and verification of our modelling results *(i.e., the multiphysics model developed and used to estimate the performance of the proposed design*). As mentioned previously, we first developed a simple y-channel microfluidic device with square *“in fluid”* electrodes. From this point, we will refer to this study as “the simple square in fluid electrode design” which is identical to the design reported in the previous reported works of Piacentini et al. [44] and Tornay et al. [45]. Section 3.1.1 provides a discussion of the results obtained from our developed multiphysics model for predicting the flow field, electric potential, and particle trajectories in the simple square electrode design.

#### 3.1.1. Verification of Computational Modelling through Model Comparisons

**Figure 6:**
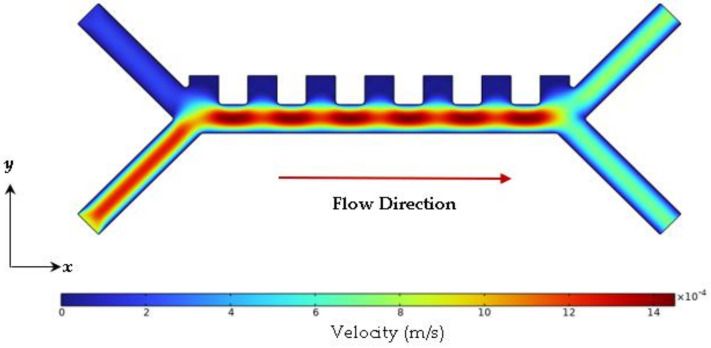
Contour of steady fluid flow through the y-channel device.

The discussion of results begins with analyzing the fluid flow through the microfluidic channel of the “simple square in fluid electrode design” as shown below in the color contour provided in Figure 6 below. As shown below in Figure 6, the fluid flow is steady from both inlets and becomes unsteady (*not to be confused with turbulence, but unorganized between electrodes 1 through 7*) as fluid travels through the region where the electrodes are exposed to the fluid, RBCs, and platelets.

**Figure 7:**
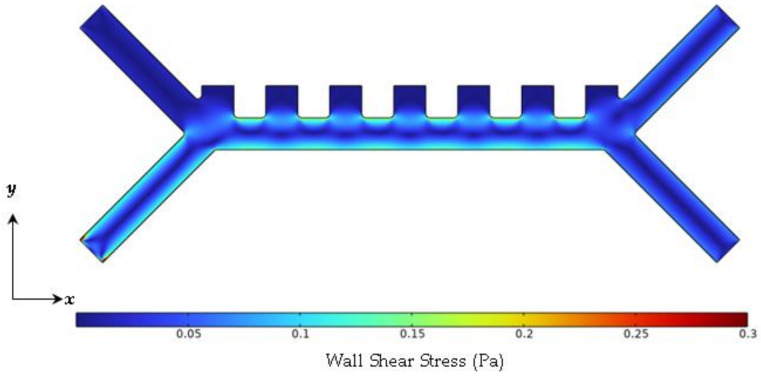
Contour of wall shear stress.

The essential argument is that although the fluid flow is unsteady, it provides a smooth transition for both RBCs and platelets to be trajected from the inlets to the outlets. One major concern in terms of unsteadiness is the level of wall shear stress that is promoted due to flow disturbance. RBCs are known to be damaged at high levels of shear rates and wall shear stresses. The threshold shear rate and wall shear stress for RBCs is 150 Pa (1500 dynes/cm^2^) for wall shear stress [46]. Figure 7 shows the wall shear stresses generated in the microfluidic chamber as a result of the fluid flow.

**Figure 8:**
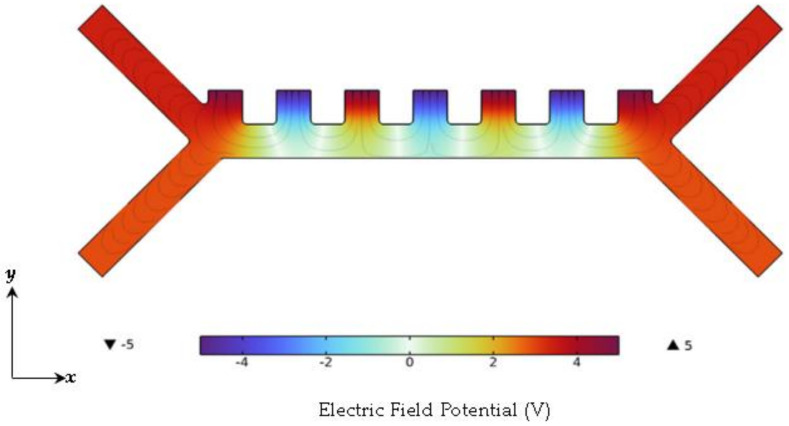
Contour of electric potential voltage.

**Figure 9:**
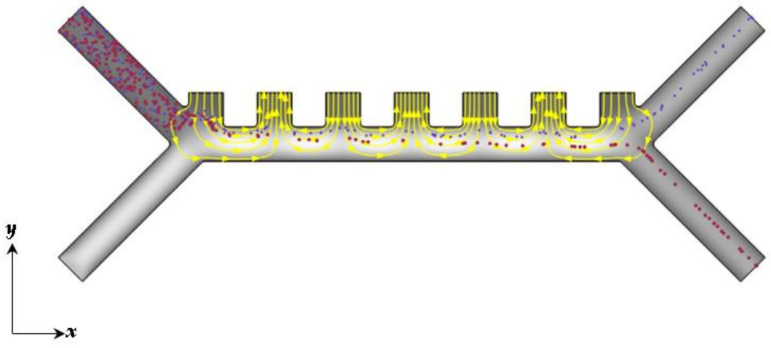
Particle trajectories with DEP force applied. The RBCs are displayed in red and the platelets in blue. The electric field lines are emphasized by the yellow streamlines. The relative size of the RBCs has been divided by two.

As shown in Figure 7, the maximum wall shear stress observed in the channel is 0.3 Pa. The design of this device would not promote large enough wall shear stresses to damage RBCs or platelets. Figure 8 provides a contour of the electric field potential inside the microchannel device. The range of voltage is −5 V to 5 V. When the field is turned off, no DEP force exists, and the red blood cells and platelets follow the same path and exit through the same outlet, as shown in Figure 9.

When a field is applied, a DEP force is present and the RBCs and platelets are separated due to the differences in their dielectric properties. This phenomenon is illustrated in Figure 9. Figure 9 provides an illustration of the platelets and RBCs entering the inlet of the simple square electrode design. In comparing the results of our developed simple square in fluid electrode design with the work of Piacentini et al. [44] and Tornay et al. [45], our work was found to be in 98% agreement in terms of throughput and potential cell purity, particle tracking, and electric field potential.

### 3.2. Simulation Results for the Saw-Tooth Electrode Design

This section of the paper presents the results from the multiphysics simulation of the proposed design.

#### 3.2.1. Mesh Independent Study

**Figure 10:**
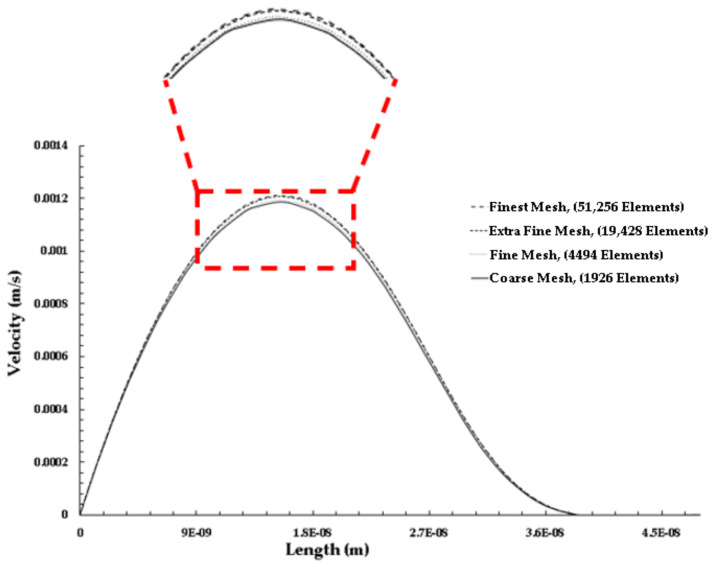
Plot of the velocity profile at the center of the device.

A mesh independence study was conducted on the proposed design to obtain the optimum mesh element size for running the simulations in which the results are more dependent on the mesh size. In this work, four types of mesh types were analyzed: coarse (*1926 elements*)*,* fine (*4494 elements*)*,* extra fine (*19,428 elements*)*,* and finest (*51,256 elements*) using a triangular and boundary layer mesh element type. A velocity and voltage potential profile extraction was used to verify the mesh size independence. A plot of the velocity profile extraction is shown below in Figure 10.

**Figure 11:**
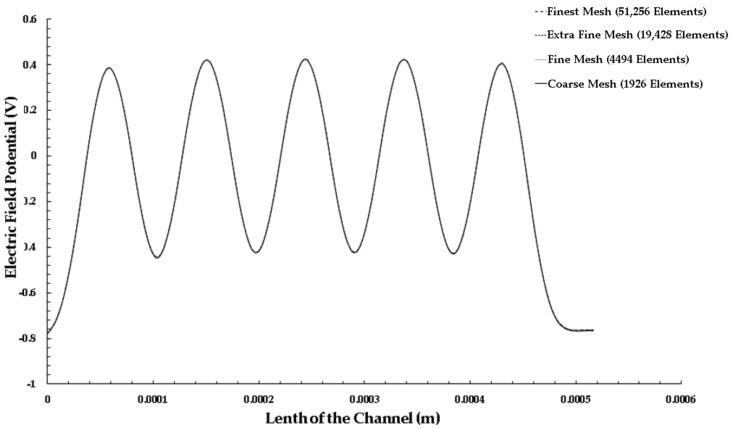
Plot of the velocity profile at the center of the device.

As shown in Figure 10, the flow profile for all meshes were fully developed and laminar. The profile is mostly parabolic, except for the regime beginning beyond 270 µm. This is due to the fluid region extending into the saw-tooth profile region. When evaluating mesh independence, it was determined that mesh independence was achieved at the “Extra Fine Mesh” setting for COMSOL that resulted in a total of 19,428 mesh elements. This resulted in less than a 5% difference in velocity results with comparing the “Extra Fine Mesh” to the “Finest Mesh” result. When comparing to the “Fine Mesh”, a less than 5% change in velocity result was observed. For computational expense, the “Extra Fine Mesh” would suffice. We also verified mesh independence through plotting the electric potential in the center of the microchannel as shown below in Figure 11.

**Figure 12:**
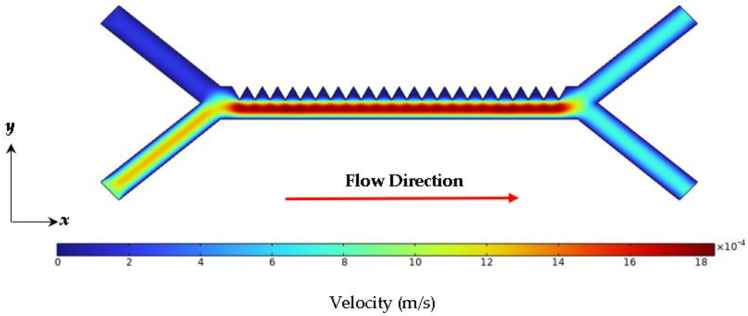
Steady flow of the fluid medium through the microfluidic device channel.

As shown in Figure 11, the plots of Coarse, Fine, Extra Fine, and Finest Mesh appear to lie on top of each other. As a result, this further verifies that the model is mesh independent at the “Extra Fine Mesh” setting. We did, however, run all simulations on the “Finest Mesh”. The rationale for this was that we wanted to have enough mesh elements to provide sufficient resolution for particle tracing. Figure 12 shows the velocity field contour in the microchannel.

**Figure 13:**
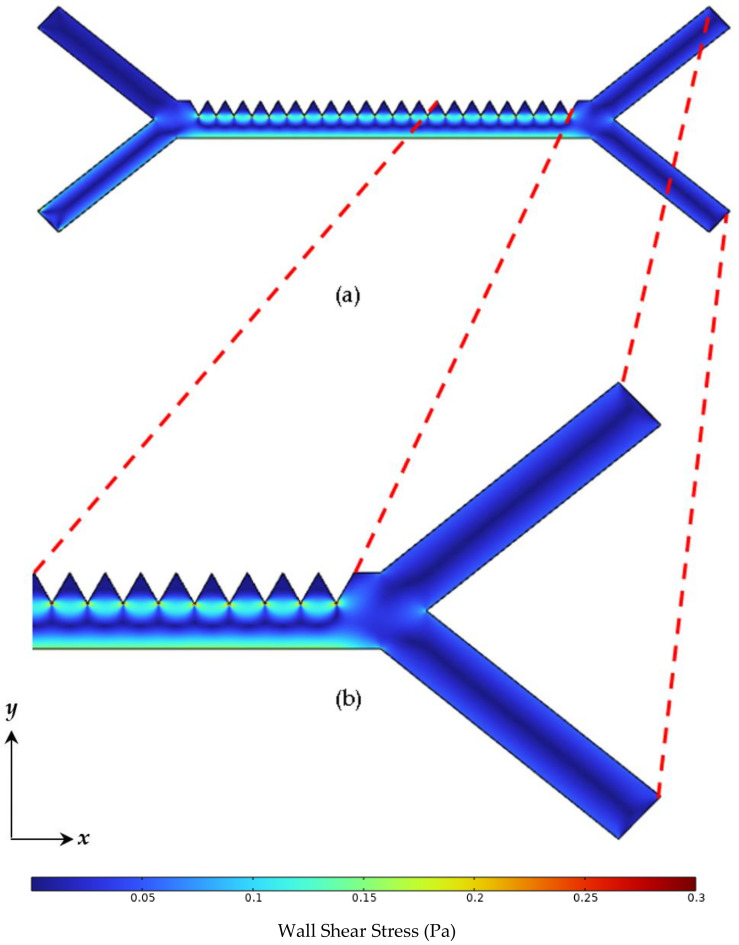
Contour of wall shear stress: (**a**) Full view of the microchannel and (**b**) zoomed in view of the microchannel and electrode region on the right end of the device.

Similar to the simple square electrode design, the fluid flow in the proposed design microchannel is steady from both inlets and becomes unsteady (*not to be confused with turbulence, but unorganized between electrodes 1 through 11*) as fluid travels through the region where the electrodes are exposed to the fluid, RBCs, and platelets. Figure 13 provides a snapshot of the wall shear stress contour.

**Figure 14:**
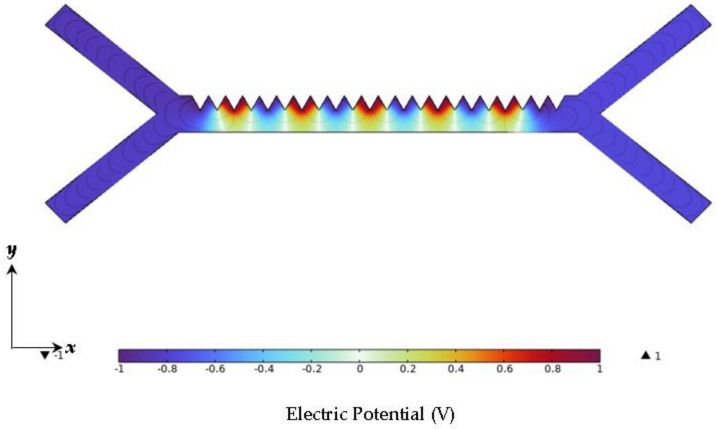
Contour of electric field potential and streamlines.

Similar to the simple square electrode design, the key argument here is that although the fluid flow is unsteady, it provides a smooth transition for both RBCs and platelets to be trajected from the inlets to the outlets. A major concern in terms of unsteadiness is the level of wall shear stress that is promoted due to flow disturbance caused by the saw-tooth electrode profile. As mentioned previously, RBCs are known to be damaged at high levels of shear rates and wall shear stresses. The contour shown in Figure 13 suggests that the proposed design does not promote high enough levels of wall shear that would damage the RBCs injected into the device. Figure 14 shows the electric field potential generated in the microfluidic device at a voltage setting of 1 V.

**Figure 15:**
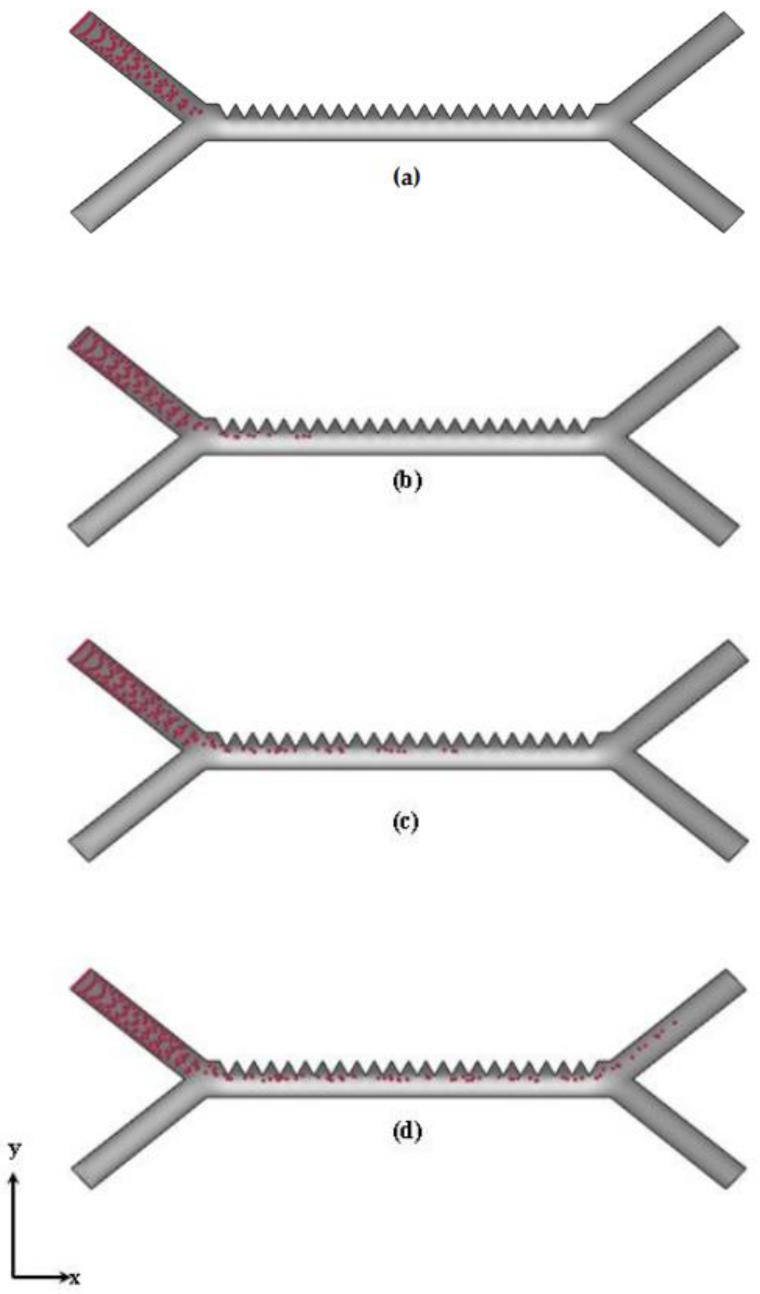
RBC and platelet continuous flow tracking with the electrodes turned off at times: (**a**) t = 1 s, (**b**) t = 1.25 s, (**c**) t = 1.5 s, (**d**), t = 2 s. As mentioned previously, for sake of visualization, the relative size of the RBCs has been divided by two.

**Figure 16:**
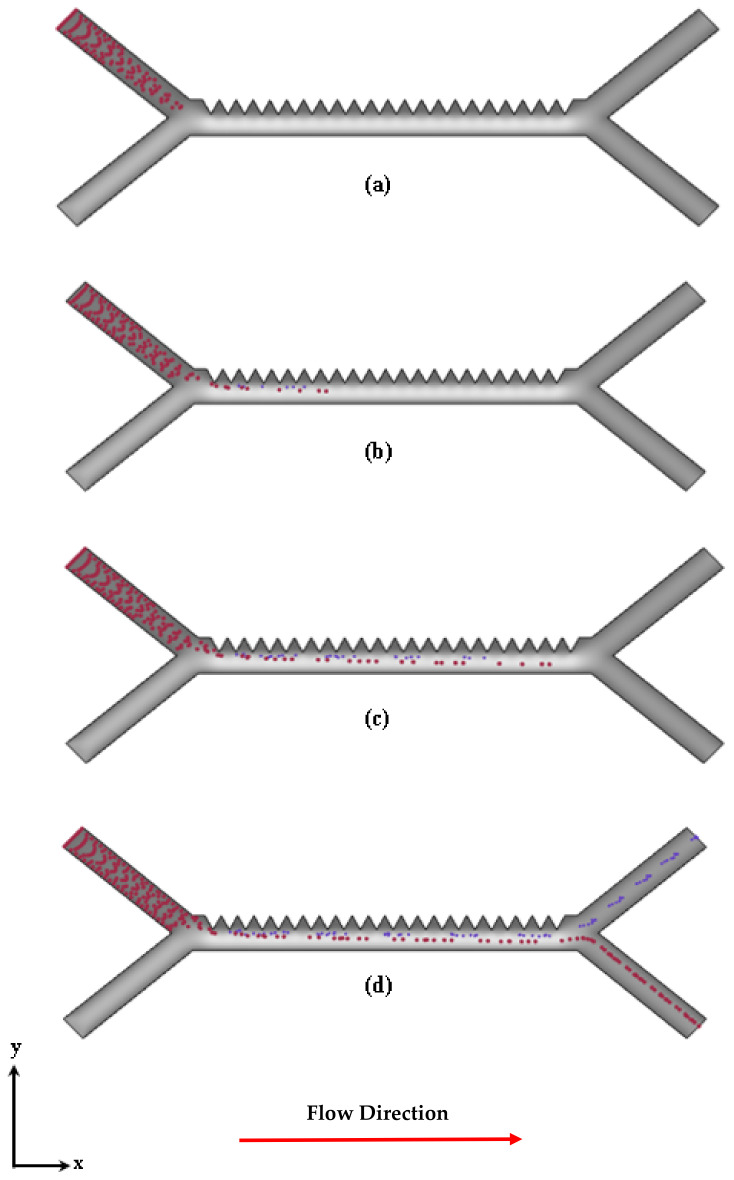
RBC and platelet continuous flow separation tracking at times: (**a**) t = 1 s, (**b**) t = 1.25 s, (**c**) t = 1.5 s, (**d**), t = 2 s.

As shown in Figure 14, the electric potential field distribution within the microchannel appears to be non-uniformly distributed through the microchannel. The saw-tooth profile appears to provide an electric potential distribution that is non-uniformly distributed throughout the microchannel from the inlet to the outlet section. This electric potential behavior should contribute to a smooth and efficient separation of RBCs from the platelets upon entry of the microchannel from the inlet. The separation behavior can be characterized by contours of the particle tracking in the microchannel as a function of time. Figure 15 and Figure 16 provide an illustration of the particle tracking within the microchannel when the electrodes are turned off and on.

Figure 15 shows contours of the particle tracking in the microchannel as a function of time while the electric field is turned off. As expected, the RBCs and platelets enter the microchannel and move throughout the microchannel under the influence of the hydrodynamic force produced by the inlet cell and buffer fluid flow. The cells are injected at a total time of 3 s with a timestep of 0.05 s. When the field is turned off, the RBCs and platelets move in the same trajectory path from the inlet to the upper outlet of the device. It takes a total time of 2–3 s from the time the cells enter through the upper left inlet of the device to move through the channel and out through the upper right outlet. Figure 16 shows contours of the particle tracking in the microchannel as a function of time while the electric field is turned on at 1 V. Similar to when the field is off, the RBCs and platelets enter and move through the microchannel under the influence of the hydrodynamic force produced by the inlet cell and buffer flow in addition to the DEP force produced by the electric field potential. Contrary to when the field is turned off, the DEP force influences the RBC to move faster and separate from the platelets as shown in Figure 16b–d. As demonstrated in Figure 16d, it takes a total time of 2 s for the RBCs and platelets to separate and exit through the outlets of the device.

**Figure 17:**
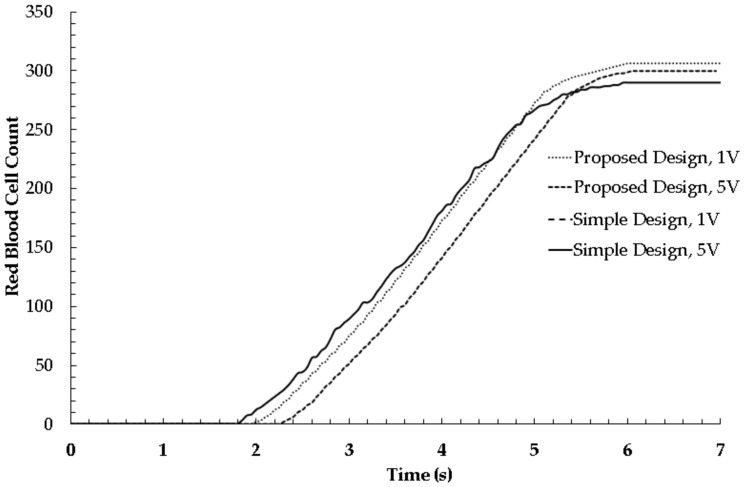
Plot of RBC cell count vs. time for varying input voltage for the proposed and simple square electrode design. Count measurements are taken in reference to the inlet and outlet.

**Figure 18:**
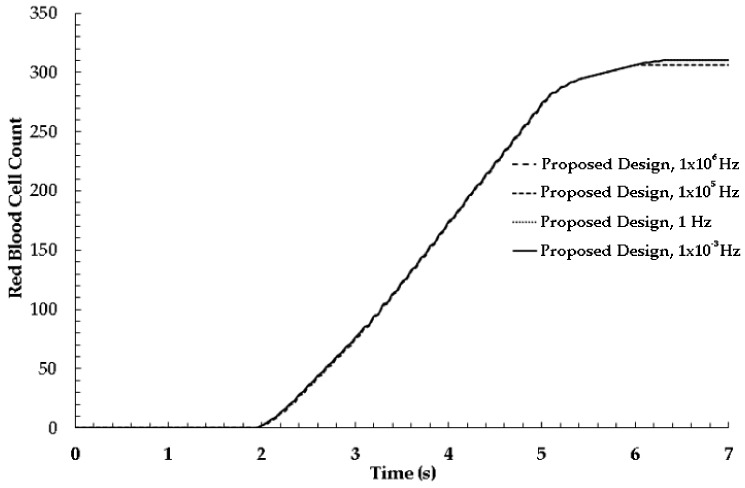
Plot of RBC cell count vs. time for varying frequency for the proposed design. Count measurements are taken in reference to the inlet and outlet.

To analyze the robustness of the proposed system design, a small statistical analysis of the RBC count and separation distance from the platelets was performed using the same concentration in RBCs and platelets while varying the DEP voltage and frequency. The performance of the proposed design was compared to the simple square electrode design. Figure 17 and Figure 18 show plots of the RBC cell count as a function of time for varying the input voltage and frequency.

As shown in Figure 17, the simple design performs the best at an optimal input voltage of 5 V. The proposed design operates efficiently at an input electrode voltage of 1 V. The RBC count increases with applied voltage and produces efficient separation efficiency at a smaller time compared to the conventional simple square electrode design.

Figure 18 shows a plot of the RBCs count vs. time for varying frequencies. From Figure 18, the proposed design operates best at a lower frequency of 1 Hz.

**Figure 19:**
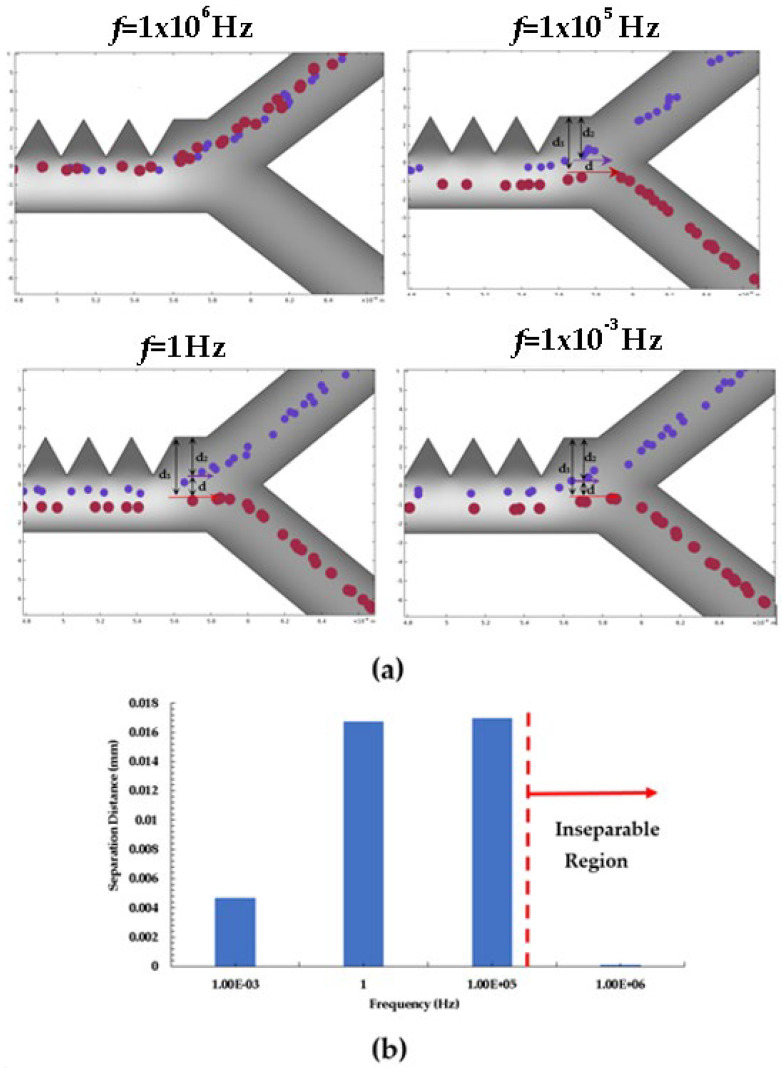
Effects of frequency on RBC separation: (**a**) Separation contours under different driving frequencies of 1 × 10^−3^ Hz, 1 Hz, 1 × 10^5^ Hz, and 1 × 10^6^ Hz. Blue color arrows mean the flow trajectory of the platelet and red color arrows mean the RBC flow trajectory. (**b**) Comparison of RBC and platelet separation distances with different driving frequencies. The relative size of the RBCs has been divided by two.

Figure 19 shows the separation performance with respect to the driving frequency at a constant driving voltage of 1 V. The distance between the RBCs and the platelets is d, and the distance from the RBC to the top wall is d = d_1_ − d_2_, where d_1_ is the distance from the RBC to the platelet and d_2_ is the distance of the platelet from the top wall. The results shown in Figure 4 indicate that the separation performance was inversely proportional to the driving frequency. That is, lower driving frequency of 1 × 10^−3^ to 1 Hz led to a better separation effect as indicated by a larger separation distance and the effect diminished as frequency increased until a failure was obtained at the 1 × 10^6^ kHz inseparable region.

Figure 19 shows that the separation distance is the greatest at 1 × 10^5^ Hz, while sufficient separation distance can also occur at frequencies as low as 1 Hz. Frequencies higher than 1 × 10^5^ Hz result in a lower separation distance to the cells being inseparable at frequencies as high as 1 × 10^6^ Hz. The performance observed with the proposed design as compared to the simple electrode design suggests that the proposed design could potentially achieve a separation efficiency and purity as high as (*99%*)*,* which is better than the reported state-of-the-art in previous works [47,48].

## 4. Conclusions

This paper presents computational results of a multiphysics simulation modelling study evaluating continuous separation of RBCs and platelets in a microfluidic device design with saw-tooth profile electrodes via DEP. The theoretical cell particle trajectory and particle separation distance study results reported in this work were predicted using COMSOL v6.0 Multiphysics simulation software. We validated the numerical model used in this work for the reported device design by developing a simple y-channel microfluidic device with square *“in fluid”* electrodes similar to the design reported previously in other works. We then compared the obtained simulation results for the simple y-channel device with the square in fluid electrodes to the reported experimental work done on this simple design which resulted in 98% agreement.

The saw-tooth electrode profile appears to be an improvement over existing designs in that the sharp corners reduced the required horizontal distance needed for separation to occur and contributed to an asymmetric DEP electric field. The results of this simulation study further suggest that this DEP separation technique may potentially be applied to improve the efficiency of separation processes of biological sample scenarios and simultaneously increase the accuracy of diagnostic processes via cell counting and sorting. The design reported in this work is an improvement over existing designs in that it can perform rapid separation of RBCs (*estimated 99% purification*) and platelets in less than a total time of 6–7 s at a minimum voltage setting of 1 V and at a minimum frequency of 1 Hz. The threshold for efficient separation of cells was observed to end at 1000 kHz for a 1 V setting. The main contributions of this work are as follows.

1. The design reported in this work is an improvement over existing designs in that it can perform rapid separation of red blood cells (*estimated 99% purification*) of platelets in less than a total time of 6–7 s at a voltage setting of 1 V and at a minimum frequency of 1 Hz.
2. The presentation of an operating parameter optimization study on driving parameters such as frequency and voltage settings.
3. The results of the simulation study suggest that the saw-tooth electrode design appears to be an improvement over existing designs in that the sharp corners reduced the required horizontal distance needed for separation to occur and contributed to a non-uniform DEP electric field.
4. The results of this simulation study suggest that this DEP separation technique may potentially be applied to improve the efficiency of separation processes of biological sample scenarios and simultaneously increase the accuracy of diagnostic processes via cell counting and sorting.

Future work will include:

1. Validating and verifying the proposed design in this work via cell flow experiments.
2. Simulating design changes such as electrode dimensions and shape, electrode space dimensions, inlet velocity ratios, driving frequency, driving voltage, and outlet separation degree angle to find the optimum design specs for cell separation efficiency.
3. Investigating the efficacy of the proposed design for cell separation efficiency using difference cell solutions.

## Figures and Tables

**Table 1:** Material and dielectric properties of the cells and fluid.

Properties	RBC	Platelet	Fluid
Density (kg/m^3^)	1050	1050	1060
Viscosity (cp)	-	-	1
Particle diameter (µm)	5	1.8	-
Relative permittivity (F/m)	59	50	-
Relative Conductivity (S/m)	0.31	0.25	-

## Data Availability

Data may be available upon request to the corresponding author.
